# A web-based questionnaire to evaluate risk factors to develop cow milk allergy

**DOI:** 10.1007/s00431-025-06070-3

**Published:** 2025-03-14

**Authors:** Meltem Dinleyici, Koray Harmanci, Didem Arslantas, Yvan Vandenplas, Ener Cagri Dinleyici

**Affiliations:** 1 Department of Social Pediatrics, Faculty of Medicine, Eskisehir Osmangazi University, Eskisehir, Turkey; 2 Department of Pediatric Allergy Immunology, Faculty of Medicine, Eskisehir Osmangazi University, Eskisehir, Turkey; 3 Vrije Universiteit Brussel, UZ Brussels, KidZ Health Castle, Brussels, Belgium; 4 Department of Pediatrics, Faculty of Medicine, Eskisehir Osmangazi University, TR-26040 Eskisehir, Turkey; 5 Department of Public Health, Eskisehir Osmangazi University, Eskisehir, Turkey

**Keywords:** Cow’s milk allergy, Food allergy, Microbiota, Antibiotic, Microbiome

## Abstract

Many environmental, genetic, and epigenetic variables are considered to influence the evolution of cow’s milk allergy (CMA). The gastro-intestinal microbiota may play a direct role in or inhibit tolerance development. In this study, we planned to evaluate the presence of previously identified risk factors for microbiota composition. This study used a cross-sectional electronic survey in Turkiye, utilizing a national convenience sample of 270 children with CMA, as reported by their caregivers, and 2154 healthy controls. We developed a web-based questionnaire to gather information on pregnancy and maternal-related factors, delivery mode, feeding patterns, antibiotic use, and the presence of pets in the home. The risk factors affecting CMA were maternal age (OR 0.897; 0.862–0.934, *p* < 0.01), presence of maternal allergic disorders (OR 3.070; 1.891–4.983, *p* < 0.001) and in both parents (OR 3.831; 1.202–12.210, *p* < 0.001), maternal weight at conception (OR 1.016; 1.003–1.030, *p* < 0.05), maternal weight gain during pregnancy (OR 1.033; 1.012–1.056, *p* < 0.01), (absence of a) pet at home (OR 1.394; 1.003–1.938, *p* < 0.05), intrapartum antibiotic use (OR 1.469; 1.092–1.975, *p* < 0.05), antibiotic use during the first 6 months of life (OR 1.933; 1.306–2.863, *p* < 0.001), and number of householders (OR 0.794; 0.650–0.969, *p* < 0.05). *Conclusion*: In addition to allergic disorders in parents, maternal weight and weight gain during pregnancy, intrapartum and first 6 months of life antibiotic use, and the presence of pets at home were found to be microbiota-related risk factors in children with CMA. Potential strategies related to microbiota composition may contribute positively to the disease’s development and progression.

**Table:** 

**What Is Known:** *• The gut microbiome contributes to the development of cow milk allergy, and disrupted microbiota maturation during the first year of life appears to be common in pediatric food allergies.* *• Factors that influence an infant’s microbiota within the first 1000 days and the relationship between these factors and microbiota may enhance allergy diagnosis, prevention, and treatment.* **What Is New:** *• Besides parental allergy disorders, maternal weight and weight gain during pregnancy, antibiotic use during intrapartum and first six months of life, and the presence of pets at home were identified as microbiota-related risk factors in children with CMA.*

**What Is Known:**

*• The gut microbiome contributes to the development of cow milk allergy, and disrupted microbiota maturation during the first year of life appears to be common in pediatric food allergies.*

*• Factors that influence an infant’s microbiota within the first 1000 days and the relationship between these factors and microbiota may enhance allergy diagnosis, prevention, and treatment.*

**What Is New:**

*• Besides parental allergy disorders, maternal weight and weight gain during pregnancy, antibiotic use during intrapartum and first six months of life, and the presence of pets at home were identified as microbiota-related risk factors in children with CMA.*

## Introduction

The rise in non-communicable diseases, including allergic diseases, in recent years has become worldwide an important health burden [1]. Cow’s milk allergy (CMA) is the predominant food allergy in infancy, with a prevalence ranging from 0.54 to 4.9%, contingent upon geographic location and feeding method (human milk versus formula feeding) [2]. Recent studies suggest that the gut microbiome plays a role in the development of allergic diseases, including CMA [3–5]. Advancements in microbiome research over the past two decades have revealed a potential correlation between the establishment of the microbiome during the first 1000 days of life and the onset of various non-communicable diseases [4, 6]. While evidence continues to affirm that microbe-host interactions govern immune regulation, recent data indicates that interactions with both environmental microorganisms and the human microbiota are crucial in modifying this process [7]. The microbiota can, under specific circumstances, directly contribute to the failure of tolerance development, resulting in food allergy sensitivity and dysbiosis. Conversely, it can promote the (re-)establishment of tolerance at other periods [4, 8, 9]. The composition of the infant microbiome is dynamic and strongly influenced by maternal, infant, and environmental-related factors. For breastfed infants, the mother is the most influential factor in the composition of the infant’s microbiota throughout the first 1000 days of life. Aside from the mother’s intestinal microbiota, the composition of mother’s milk and its microbial content also contribute [6, 10–13]. Antibiotics administered to the mother and/or the infant can also disrupt gut homeostasis and significantly raise the risk of allergic diseases [4, 14, 15].

Disruption of the integrity and function of the epithelial barrier is a consequence of dysbiosis [16]. Research indicates that the microbiota significantly contributes to maintaining the integrity of the mucosal epithelial barrier, which in turn plays a crucial role in immune system regulation, dysregulation, and the development of allergies. Infants with CMA exhibit variations in symptoms, disease progression, and in the acquisition of tolerance [17]. Many environmental, genetic, and epigenetic variables are considered to influence the evolution of CMA [18, 19]. Hoskinson and s colleagues [20] looked at early-life factors and microbiome features in 1115 children who were part of the CHILD study birth cohort. At age 5, these factors consistently linked to four different allergic diagnoses: atopic dermatitis, asthma, food allergies, and allergic rhinitis. They collected extensive data, including familial demographic data, mode of delivery, birth weight, breastfeeding, antibiotic use, and detailed allergy evaluation. They showed that impaired first-year microbiota maturation seems universal to pediatric allergies, including food allergies. They showed a decrease in gut microbiome maturation, within the alteration of a core group of species, functional pathways, and metabolic imbalance associated with reduced microbiota-maturation age and elevated risk of allergy. They highlighted that microbiota maturation during the early period of life provides a focal point to identify deviations from normative development to predict and prevent allergic disease [20]. Better understanding of the factors that influence an infant’s microbiota during the first 1000 days and the correlation between these factors and microbiota disorders could improve allergy diagnosis, prevention, and treatment. Currently, there is no effective treatment available for FA, with avoidance being the primary measure (17). In this study, we planned to evaluate the presence of previously identified risk factors for dysbiosis in children with CMA and compare them to healthy children.

## Methods

The microbalance study is a project evaluating microbiota composition and associated factors in healthy and fault children. In this part, we developed a self-completion web-based questionnaire for parents with the intention to determine risk factors related to microbiota composition in their children. We created a web-based questionnaire via SurveyMonkey, which included 70 questions relevant to previously described microbiota-related risk factors, including maternal factors during pregnancy, delivery mode, breastfeeding, and complementary feedings. Parents who have a child with physician-diagnosed CMA responded to 30 additional questions about CMA symptoms, signs, and follow-up. We sent an electronic cover letter and questionnaire via SurveyMonkey to a convenience sample of participants, which included parents of both children with CMA and healthy children. We mailed the questionnaire along with a cover letter that outlined the study details, emphasized its purpose and confidentiality, and reminded potential participants that their participation was voluntary. The study did not provide any financial incentives for participation. The Eskisehir Osmangazi University Faculty of Medicine Local Ethical Committee (01.06.2021/08) approved this study. The procedures conducted in this trial adhered to the ethical criteria set by the institutional and/or national research committee, as well as the 1964 Helsinki Declaration and its subsequent revisions or similar ethical standards. All participants confirmed their agreement to participate in the study before responding to the online questionnaire.

### Inclusion and exclusion criteria

Parents (mother or father) who have children with physician-confirmed CMA or healthy children between 6 and 120 months and agreed to participate in this study. For healthy children, the exclusion criteria include the presence of chronic disease, chronic medication, and relevant surgical interventions. We exclude questionnaires not properly filled out.

### Questionnaire

The questionnaire encompassed questions related to the following topics: demographic variables of the child, such as age, gender, and city of residence; and parental demographic variables, such as age, educational status, number of children, and allergic disorders. The questionnaire also inquired about the house and living conditions, including the presence of a garden, the number of rooms, the number of householders, smoking status, and the presence of pets such as cats and dogs, during pregnancy and beyond. We also collected information on maternal diseases like gestational diabetes, hypertension, urinary tract infections, the use of antibiotics during pregnancy, the mother’s weight and height at conception, her weight at delivery, and the absolute and percentage weight gain during pregnancy. Other factors include delivery mode (vaginal or C-section), the type of C-section (emergency or elective), and the use of antibiotics intrapartum or during the first 7 days after delivery; gestational age, birth weight, and antibiotic use during the first 1 month and 6 months of life; exclusive breastfeeding during the first 6 months of life; formula feeding; probiotic use; and the reason for probiotic use.

### CMA-related questions

If the parents answered affirmatively to the question “Does your child have an allergy to cow’s milk, and has this diagnosis been confirmed by a physician?” they are asked to answer additional questions regarding the symptoms, signs, and follow-up of CMA. This section of the questionnaire contains questions regarding the symptoms associated with CMA, such as vomiting, diarrhea, presence of blood or mucus in stool, constipation, skin abnormalities (including eczema), respiratory system symptoms, anaphylaxis or angioedema, anal lesions, failure to gain weight, and colic or abdominal pain. It also includes inquiries about the presence of other concurrent conditions, such as infantile colic, atopic dermatitis, allergic rhinitis, asthma, celiac disease, frequent infections (more than 8 per year), and constipation. Information is also collected regarding nebulization treatment, a specific CMA formula, and if an epinephrine autoinjector was prescribed.

### Statistical analysis

We sent 3200 questionnaires via SurveyMonkey for this study. In 2601 of these questionnaires, the responses were adequate to perform further analysis. Parents of healthy children completed 2154 of these questionnaires. 447 parents reported that their child had a “food allergy”. After evaluating 447 questionnaires, we found that 270 of them had an only cow’s milk allergy, which we then included in the analysis. We excluded 177 questionnaires due to non-cow’s milk food allergies or multiple food allergies. In total, 2601 parents responded to the questionnaire. We conducted the final analyses on 270 children diagnosed with CMA and 2154 children presumed to be healthy. We used the SPSS Statistical Package for the Social Sciences (Illinois, CA, USA) for the statistical analysis. We depicted the quantitative variables using the mean value plus or minus the standard deviation for normally distributed data, or the median with the interquartile range for non-normally distributed data. We used independent *t*-tests to compare continuous data according to a normal distribution and applied Mann–Whitney *U* tests for data that deviated from normality. We evaluated the associations among qualitative factors with a chi-square test. We computed the odds ratios (OR) for the CMA group, utilizing the healthy group as the reference cohort. We also used stepwise logistic regression and a likelihood ratio test to build a model and examine CMA risk factors. We considered a *p*-value less than 0.05 as statistically significant.

## Results

This study included 270 children diagnosed with CMA. Signs of CMA that were present at diagnosis were vomiting in 74 cases (27.4%), diarrhea (*n*:76; 28.1%), blood or mucus in the stool (*n*: 152; 56.3%), constipation (*n*:24; 8.9%), skin problems (including eczema) (*n*: 168; 62.2%), respiratory symptoms including wheezing (*n*:46; 17%), anaphylaxis or angioedema (*n*: 5; 1.9%), anal lesions (*n*: 7; 2.6%), faltering growth (*n*: 4; 1.5%), infantile colic (*n*:73, 27%) and 12 (4.4%) had frequent infections. The majority of the mothers (*n*:206; 76.3%) adhered to a dietary regimen. 45 patients, or 16.7% of the total, received prescriptions for Epinephrine autoinjectors were prescribed for 45 children (16.7% of the total).

### Comparison of microbiota-related risk factors between children with CMA and healthy children

A comparison of the microbiota-related traits of the 270 children CMA (151 boys and 110 girls) with those of the 2154 healthy children (1125 boys and 1029 girls) is shown in Table 1. There is no statistically significant difference in age and gender distribution (*p* > 0.05) of the children. The maternal and paternal age of children were lower in the CMA group than in the control group (*p* < 0.001 for both). The prevalence of allergic disorders was significantly greater among both parents in the group with CMA compared to the group of healthy children (*p* < 0.001 for all). The number of children and household members at home was lower in the CMA group (*p* < 0.001 for both). The proportion of children residing in an apartment was greater in the CMA group compared to the healthy children (*p* < 0.05). The number of rooms and the percentage of a garden at home were both lower in the CMA group. The father’s smoking status is significantly greater in children with CMA compared to healthy children (*p* < 0.05). Throughout pregnancy, the prevalence of pets such as cats, dogs, or birds was comparable across all groups. The presence of pets, specifically cats, at home after pregnancy was higher in the CMA group compared to the control group (*p* = 0.001).

**Table 1:** Comparison of demographical and presence of microbiota-related risk factors between children with CMA and healthy children

	CMA (*n* = 270)	Healthy children (*n* = 2154)	OR 95%CI; *p*
Age (months)	39.4 ± 31.9	41.8 ± 32.2	ns
Gender (boys/Girls)	151/119	1125/1029	ns
Mother age (years)	34.7 ± 6.1	39.7 ± 5.9	***p *< 0.001**
Father age (years)	37.7 ± 6.4	42.4 ± 6.6	***p*** **< 0.001**
Presence of allergic disorders			
Mother *n* (%)	45 (16.7)	100 (4.6)	**OR 4.105 95%CI 2.746–6.065; *p*** **< 0.001**
Father *n* (%)	18 (6.7)	45 (2.1)	**OR 3.345 95%CI 1.794–6.004 ; *p *< 0.001**
Both parents *n* (%)	11 (4.1)	7 (0.3)	**OR 12.999 95%CI 4.556–39.903; *p *< 0.001**
Number of children at home	1.37 ± 0.5	1.54 ± 0.6	***p *< 0.001**
Number of household members	2.35 ± 0.7	2.57 ± 0.7	***p *< 0.001**
Number of rooms	3.56 ± 0.7	3.67 ± 0.8	***p *< 0.05**
Presence of garden	171 (63.3)	1593 (74.0)	**OR 0.607 95%CI 0.462–0.801; *p *< 0.001**
Mother smoking status	58 (21.5)	547 (25.4)	ns
Father smoking status	107 (39.6)	722 (33.5)	**OR 1.302 95%CI 0.994–1.700; *p *< 0.05**
Smoking status at home	144 (53.3)	1162 (53.9)	ns
Presence of pet during pregnancy			
Cat	16 (5.9)	120 (5.6)	**ns**
Dog	10 (3.7)	55 (2.6)	ns
Bird	10 (3.7)	80 (3.7)	**ns**
None	234 (86.7%)	1899 (88.1%)	**ns**
Presence of pet after pregnancy			
Cat	23 (8.5)	340 (15.8)	**OR 0.497 95%CI 0.304–0.777; *p *< 0.01**
Dog	10 (3.7)	141 (6.5)	ns
Bird	18 (6.7)	224 (10.4)	ns
None	219 (81.1%)	1449 (67.2%)	**OR 1.897 95%CI 1.407–2.582; *p *< 0.001**
Disorders during pregnancy			
Gestational diabetes	23 (8.5)	164 (7.6)	ns
Hypertension	8 (3.0)	65 (3.0)	ns
Urinary tract infections	43 (15.9)	147 (6.8)	**OR 2.585 95%CI 1.747–3.765; *p* < 0.001**
Antibiotic usage during pregnancy	49 (18.1)	183 (8.5)	**OR 2.387 95%CI 1.654–3.399; *p *< 0.001**
At first day of pregnancy			
Maternal weight (kg)	62.4 ± 10.8	60.9 ± 10.3	***p *< 0.05**
Maternal BMI (kg/m^2^)	23.0 ± 3.9	22.5 ± 3.69	ns
Overweight	47 (17.9)	312 (14.9)	**ns**
Obesity	16 (6.1)	102 (4.9)	**ns**
Maternal diet preference			
None	241 (89.2)	1846 (85.7)	**ns**
Gluten free	6 (2.2)	40 (1.9)	**ns**
Intermittent fasting	14 (5.2)	137 (6.4)	ns
Vegan/vegetarian	9 (3.3)	29 (1.3)	**ns**
Weight gain (kg) during pregnancy	15.5 ± 6.32	14.6 ± 6.61	***p *<0.01**
Weight gain (%) during pregnancy	25.8 ± 11.3	24.9 ± 12.0	***p *<0.05**
Maternal weight at labor	77.9 ± 11.3	75.4 ± 11.2	** < 0.001**
Delivery mode			
Vaginal delivery	61 (22.6)	484 (22.4)	ns
C-section	209 (77.4)	1670 (77.5)	ns
Emergency C-section	80 (29.6)	546 (25.3)	ns
Elective C-section	129 (47.8)	1124 (52.2)	ns
Intrapartum or first 7 days antibiotic use	113 (41.9)	590 (27.4)	**OR 1.904 95%CI 1.454–2.487; *p *< 0.001**
Birth weight (gram)	3357 ± 541	3250 ± 562	***p *< 0.01**
Prematurity	51 (18.9)	517 (24.0)	ns
Breastfeeding during first 24 h of life	242 (89.6)	1924 (89.3)	ns
Exclusive breastfeeding during first 6 months of life	110 (40.7)	898 (41.7)	ns
Formula feeding	160 (59.3)	1256 (58.3)	ns
Formula feeding at first months of life	96 (35.6)	657 (30.5)	ns
Formula feeding at first 4 months of life	129 (47.7)	983 (45.6)	ns
Formula feeding at 6 months of life	158 (58.5)	1202 (55.8)	ns
Antibiotic use at first month of life	103 (39.0)	393 (19.2)	**OR 2.681 95%CI 2.023–3.543; *p* < 0.001**
Antibiotic use at first 6 months of life	151 (57.2)	653 (31.9)	**OR 2.839 95%CI 2.170–3.722; *p* < 0.001**

During pregnancy, there was no significant difference in the occurrence of gestational diabetes and hypertension across the groups (*p* > 0.05). Nevertheless, urinary tract infections and the use of antibiotics for any reason during pregnancy were more prevalent in children with CMA compared to healthy children (*p* < 0.001).

Maternal weight was significantly higher in mothers of children with CMA compared to mothers of healthy children at the beginning of pregnancy (*p* = 0.036). The maternal BMI and maternal overweight/obesity status did not differ significantly across the groups (*p* > 0.05). The average weight gain during pregnancy, the percentage of weight gain during pregnancy, and the final weight of mothers at the time of labor were all higher in mothers of children with CMA compared to mothers of healthy children (with *p*-values of < 0.032, < 0.033, and < 0.001, respectively). The rates of vaginal delivery, or C-section, were similar between the two groups. Between the groups, the rates of emergency C-section and elective C-section were comparable, with no statistically significant difference (*p* > 0.05).

Healthy infants had a higher incidence of prematurity (*p* = 0.034), and children with CMA had significantly higher birth weights compared to healthy children (*p* = 0.03). The use of antibiotics during labor and delivery, as well as during the first week after giving birth, was considerably greater in the group of mothers with a history of chronic medical conditions compared to the group of healthy mothers (*p* < 0.001). The use of antibiotics during the first months of infancy, as well as the first 6 months of life, is more common in children with CMA compared to healthy children (*p* = 0.0024 and *p* = 0.0001, respectively). The rates of breastfeeding initiation during the first 24 h of birth and exclusive breastfeeding for the first 6 months of life were comparable among the groups. The rates of formula feeding during the initial months of life, specifically the first 4 and 6 months, did not differ significantly across the groups (*p* > 0.05).

Logistic regression analysis was performed in the model created with the variables of mother’s and father’s age, presence of maternal allergic disorders, presence of allergic disorders in both parents, maternal urinary tract infections during pregnancy and antibiotic use, maternal weight at conception, intrapartum antibiotic use, antibiotic use during the first and first six months of life, presence of a pet at home, and number of householders to determine the factors affecting CMA risk. The risk factors affecting CMA were maternal age (OR 0.897; 0.862–0.934, *p* < 0.01), presence of maternal allergic disorders (OR 3.070; 1.891–4.983, *p* < 0.001), presence of allergic disorders in both parents (OR 3.831; 1.202–12.210, *p* < 0.001), maternal weight at conception (OR 1.016; 1.003–1.030, *p* < 0.05), maternal weight gain during pregnancy (OR 1.033; 1.012*–*1.056, *p* < 0.01), no pet at home (OR 1.394; 1.003–1.938, *p* < 0.05), intrapartum antibiotic use (OR 1.469; 1.092–1.975, *p* < 0.05), antibiotic use during the first 6 months of life (OR 1.933; 1.306–2.863, *p* < 0.001), and number of householders (OR 0.794; 0.650–0.969, *p* < 0.05).

## Discussion

This study revealed a correlation between infants with CMA and factors such as maternal age, the presence of allergic disorders in the mother or in both parents, maternal weight at conception, and weight gain during pregnancy. Also, administration of antibiotics during intrapartum or during the first 6 months of life was found to be related factors. The number of householders and the presence of pets at home also played a significant role.

Studies that looked at the relationship between changes in the microbiota’s diversity and/or function and becoming sensitive to foods early in life suggest that the microbiome may be an interfering factor with the development of food allergies, such as CMA [4, 21]. A plethora of modifiable risk factors has been identified that elevate an individual's susceptibility to food allergies, including delivery mode, baby feeding practices, maternal nutrition, antibiotic use during gestation, and rural versus urban residency [17, 22]. Contemporary research indicates that it is probably a confluence of risk factors. Both controllable and nonmodifiable risk factors affect the composition of an individual’s gut microbiota [23].

While exposure to microbes likely occurs already during pregnancy, the infant’s microbiota composition is undoubtedly influenced by maternal and pregnancy-related factors at birth [6]. Researchers have reported that excessive gestational weight gain increases the risk of childhood allergic diseases, especially asthma [24]. Our data regarding the weight of the mother endorses this hypothesis. While there are conflicting results regarding the relationship between gestational weight gain and food allergies, our study suggests that maternal weight gain appears to be related to CMA. A notable connection between maternal diet during gestation or breastfeeding and reduced risk of later allergy illness in the child was better adherence to a Mediterranean diet. Conversely, studies often, but not uniformly, indicate that a maternal diet during gestation rich in vegetable oils, margarine, nuts, and fast food elevates a child’s risk of having allergic disease [17, 25]. Research about food allergies indicates that incorporating common allergens into the maternal diet during gestation and lactation may provide protective benefits, but some trials yielded ambiguous results [17].

Children with CMA have a higher frequency of infection and use of antibiotics compared to healthy children [26]. In our study, intrapartum antibiotic use is 1.4 times higher, and antibiotic use during the first 6 months of life is 1.9 times higher in children with CMA than in the healthy group. Exposure to antibiotics during pregnancy can increase the risk for children to develop allergic disorders [27]. Both prenatal and postnatal antibiotic exposure have been linked to a heightened risk of asthma [27]. Antibiotics can modify the composition of the native microbial population, thus diminishing their variety and impacting their genetic traits and functionality [4, 27]. Regrettably, the use of intrapartum antibiotics is escalating in conjunction with the rising frequency of cesarean section deliveries [27, 28]. However, our study failed to demonstrate the association between allergic diseases and cesarean sections. Our study cohort also confirmed the high rates of cesarean sections in Turkiye [29]. Although cesarean sections did not seem to be effective, intrapartum antibiotic use was associated with them, suggesting the effect of this factor on the development of CMA. Most infants receive multiple courses of antibiotics during the first two years of life [30]. Research from human studies associates the utilization of antimicrobial drugs with a rise in food allergy prevalence, particularly during the newborn period, which is a crucial time for antibiotic exposure [4]. Love et al. demonstrated a significant increase in the likelihood to develop food allergy in children who received five or more antibiotic prescriptions in their first year of life [31]. In these cases, interventions aimed at reducing antibiotic use may have positive effects on the development of allergic disorders. Therefore, rational antibiotic use may have positive effects on the microbiota composition and may lead to changes in the disease's development and course.

Our study had some limitations. The study utilized a questionnaire that gathered information from the parents. The study included physician-diagnosed CMA cases, but the patient information was based on parental evaluations rather than on the medical records. For this reason our analysis does not include data on allergic sensitization including results from prick tests or specific IgE levels. In this study, we did not perform microbiota analysis, this is a questionnaire study about the risk factors. This is a retrospective part of the our microbalance study. We planned to perform a prospective study with these risk factors and microbiota analysis together. The study’s strength was that we reached a sufficient number of patients in the CMA and control groups, as well as patients from all regions of the country and different sociocultural levels.

Prior research indicates that early infancy, particularly between 3 and 6 months of age, is a critical period during which gut microbiota influences the development of food allergies [17]. Nonetheless, it remains uncertain whether sustaining this “healthy” gut flora is sufficient to elicit tolerance to food [4]. While recent discussions have highlighted the beneficial effects of environmental regulation on microbiota restoration, our study revealed that genetic factors play a more significant role in the development of allergic disease in mothers and parents. These factors cannot be changed. Therefore, controlling other associated microbiota-related factors in children with a family history of allergy may be beneficial.

## Data Availability

The data are available from the corresponding author upon reasonable request.

## Abbreviations

CMA: Cow milk allergy
OR: Odds ratio
